# Microbiological Studies on the Influence of Essential Oils from Several *Origanum* Species on Respiratory Pathogens

**DOI:** 10.3390/molecules28073044

**Published:** 2023-03-29

**Authors:** Bartłomiej Piasecki, Viktória L. Balázs, Anna Kieltyka-Dadasiewicz, Péter Szabó, Béla Kocsis, Györgyi Horváth, Agnieszka Ludwiczuk

**Affiliations:** 1 Department of Pharmacognosy with the Medicinal Plant Garden, Medical University of Lublin, 20-093 Lublin, Poland; 2 Department of Pharmacognosy, Faculty of Pharmacy, University of Pécs, 7624 Pécs, Hungary; 3 Department of Plant Production Technology and Commodity, University of Life Sciences in Lublin, 20-950 Lublin, Poland; 4 Institute of Geography and Earth Sciences, Faculty of Sciences, University of Pécs, 7624 Pécs, Hungary; 5 Department of Medical Microbiology and Immunology, Medical School, University of Pécs, 7624 Pécs, Hungary

**Keywords:** oregano, essential oil, GC-MS, biofilm inhibition, MRSA, *Haemophilus*, *Pseudomonas*

## Abstract

Essential oils (EOs) with established and well-known activities against human pathogens might become new therapeutics in multidrug-resistant bacterial infections, including respiratory tract infections. The aim of this study was to evaluate the antimicrobial activity of EOs obtained from several samples of *Origanum vulgare*, *O. syriacum,* and *O. majorana* cultivated in Poland. EOs were analyzed by GC-MS and tested against four bacterial strains: *Staphylococcus aureus* (MRSA), *Haemophilus influenzae*, *Haemophilus parainfluenzae,* and *Pseudomonas aeruginosa*. Chemical analyses showed that the Eos were characterized by a high diversity in composition. Based on the chemical data, four chemotypes of *Origanum* EOs were confirmed. These were carvacrol, terpineol/sabinene hydrate, caryophyllene oxide, and thymol chemotypes. Thin-layer chromatography-bioautography confirmed the presence of biologically active antibacterial components in all tested EOs. The highest number of active spots were found among EOs with *cis*-sabinene hydrate as the major compound. On the other hand, the largest spots of inhibition were characteristic to EOs of the carvacrol chemotype. Minimal inhibitory concentrations (MICs) were evaluated for the most active EOs: *O. vulgare* ‘Hirtum’, *O. vulgare* ‘Margarita’, *O. vulgare* ‘Hot & Spicy’, *O. majorana*, and *O. syriacum* (I) and (II); it was shown that both *Haemophilus* strains were the most sensitive with an MIC value of 0.15 mg/mL for all EOs. *O. majorana* EO was also the most active in the MIC assay and had the highest inhibitory rate in the anti-biofilm assay against all strains. The most characteristic components present in this EO were the *trans*-sabinene hydrate and terpinen-4-ol. The strain with the least sensitivity was the MRSA with an MIC of 0.6 mg/mL for all EOs except for *O. majorana,* where the MIC value reached 0.3 mg/mL. Scanning electron microscopy performed on the *Haemophilus influenzae* and *Haemophilus parainfluenzae* biofilms showed a visible decrease in the appearance of bacterial clusters under the influence of *O. majorana* EO.

## 1. Introduction

An alarming need for new therapeutics that are active against multidrug-resistant bacterial strains has prompted researchers to study plants and herbs that are rich in essential oils (EOs), many of which have established and well-known activity against human pathogens. According to the National Library of Medicine, the number of publications containing the phrase “essential oils” is growing every year, reaching more than 2500 annually for the past 2 years. Among the most common herbs containing essential oils are aromatic plants classified in genus *Origanum*. This genus is within the Lamiaceae family and consists of 67 species according to the World Flora Online [1], but that number varies depending on different sources. Species create many subspecies and hybrids and the highest natural prevalence of these occur in the Mediterranean region where they have been used since antiquity as herbal medicines, condiments, and fragrance ingredients. Although volatile compounds, which can be obtained in the form of the essential oil, are the most abundant and desirable compounds found in *Origanum*, the phytochemistry of this genus is far more complex, and, in addition to volatiles, a wide range of phenolic compounds, e.g., phenolic acids and flavonoids, are also present [2]. The rich chemical composition of oregano determines its various pharmacological activities. In folk medicine oregano is mainly used to treat respiratory disorders and dyspepsia. Among other biological activities, antidiabetic, antiproliferative, vasoprotective, anti-inflammatory, antibacterial, and antiviral properties are worth mentioning [2,3,4,5,6,7]. The diverse biological activities of the plant were primarily found to be due to *Origanum* essential oil constituents. The composition of EOs varies considerably depending on the species and collection place, but also by the method and conditions of essential oil distillation. It is well known that within oregano, different chemotypes can be defined on the basis of a single prominent compound or group of compounds. For example, there are carvacrol, caryophyllene oxide, γ-terpinene, *trans*-sabinene hydrate, and thymol, among others [8,9,10].

The antibacterial activity of oregano is extensively described in the literature showing a wide range of microorganisms that are sensitive to either EOs or their single compounds, mainly carvacrol and thymol [11,12]. It has also been reported that EOs containing aldehydes or phenols, such as cinnamaldehyde, neral, geranial, carvacrol, or thymol as the major components, show high antibacterial activity [13,14,15]. Some studies have demonstrated that whole EOs usually have higher antibacterial activity than a mixture of their major components, suggesting that the minor components are critical in synergistic activity [13,16].

The ability of pathogenic strains to create biofilms is widely regarded as one of the most important problems and hurdles in current medical practice and effective anti-infective therapy. Infections caused by these microorganisms are harder to eradicate and the cost of the treatment is increased. In biofilms, the cells have up to 1000 times greater resistance to the antimicrobial agents [17,18]. However, there is evidence of the significant inhibition of bacterial strains creating biofilm by EOs. Therefore, EOs could be an essential component in the fight against antibiotic resistance due to their efficient anti-biofilm activities [19,20,21,22,23,24].

The aim of this study was to link up the antibacterial and anti-biofilm properties of *Origanum* EOs with their chemical compositions. Among the oregano EOs examined in this work, the antimicrobial properties, chemical compositions, and antioxidant activities of *O. vulgare*, *O. vulgare* ‘Hirtum’, *O. vulgare* ‘Variegata’, *O. vulgare* ‘Hot & Spicy’, *O. syriacum,* and *O. majorana* have been studied by other researchers [25,26,27,28,29,30]. To the best of the authors’ knowledge, the antimicrobial properties of *O. vulgare* ‘Margarita’ and *O. vulgare* ‘Aureum’ were examined here for the first time. This research gives a unique insight into the chemotype-dependent bioactivity of up to nine EOs of plants representing the same genus against popular human pathogens known for problematic treatments.

## 2. Results

### 2.1. Gas Chromatography-Mass Spectrometry (GC-MS)

GC-MS analyses showed remarkable differences in the composition of the investigated EOs, and these results are presented in Table 1. 

The data presented in Table 1 show that the most characteristic component of the examined essential oils was carvacrol. This compound was the most abundant in four among nine examined EOs. These were oils obtained from the aerial parts of *O. vulgare* cultivars ‘Hirtum’ (**2**), ‘Margarita’ (**3**), and ‘Hot & Spicy’ (**4**), as well as from *O. syriacum,* denoted as (I) (**6**). The relative percentage of carvacrol in these EOs was between 58 and 86%. All of these essential oils were classified within the carvacrol chemotype. 

Essential oil (**1**) was hydrodistilled from *O. vulgare* and showed the presence of carvacrol as well as considerable amounts of sesquiterpenoids, including caryophyllene oxide and spathulenol. The relative amount of these two components was almost 27% and was higher than the carvacrol content (22%). This finding is in agreement with the previous data published by Baj and coworkers [8] and indicates that this essential oil belongs to the caryophyllene oxide chemotype. 

The major component present in EOs obtained from *O. majorana* (**5**) and *O. vulgare* cultivars ‘Variegata’ (**7**) and ‘Aureum’ (**8**) was the *trans*-sabinene hydrate. The relative percentage of this compound in *O. majorana* EO was 37%, while in both cultivars of *O. vulgare* this was below 30%. As in the case of other oregano EOs, the presence of carvacrol was also confirmed in these samples. The lowest amount of carvacrol was detected in the *O. majorana* EO (8%), while the amount of carvacrol in the EO from *O. vulgare* ‘Variegata’ was almost equal to the *trans*-sabinene hydrate. The literature data indicated that *trans*-sabinene hydrate can be partly rearranged to form terpinen-4-ol [31]. The amount of this monoterpene alcohol in the mentioned EOs was relatively higher in comparison to the other essential oils. Another terpinene-type alcohol that was characteristic for these three EOs was α-terpineol. EOs **5**, **7,** and **8** were classified in the terpineol/sabinene hydrate chemotype. 

The essential oil hydrodistilled from the aerial parts of *O. syriacum* (II) (**9**) was found to produce thymol and carvacrol as the major components. The relative percentage of both constituents was over 72%, but the dominant compound was thymol (47%). Other characteristic monoterpenoids identified in this EO were γ-terpinene and *p*-cymene. Because of the high content of thymol, EO (**9**) was classified in the thymol chemotype. 

GC-MS analysis of the chemical composition of Origanum essential oils showed that they can be divided into four chemotypes:

1. carvacrol: EO **2**, **3**, **4**, **6**
2. caryophyllene oxide: EO **1**
3. terpineol/sabinene hydrate: EO **5**, **7**, **8**
4. thymol: EO **9**

### 2.2. Thin-Layer Chromatography-Direct Bioautography (TLC-DB)

**Figure 1:**
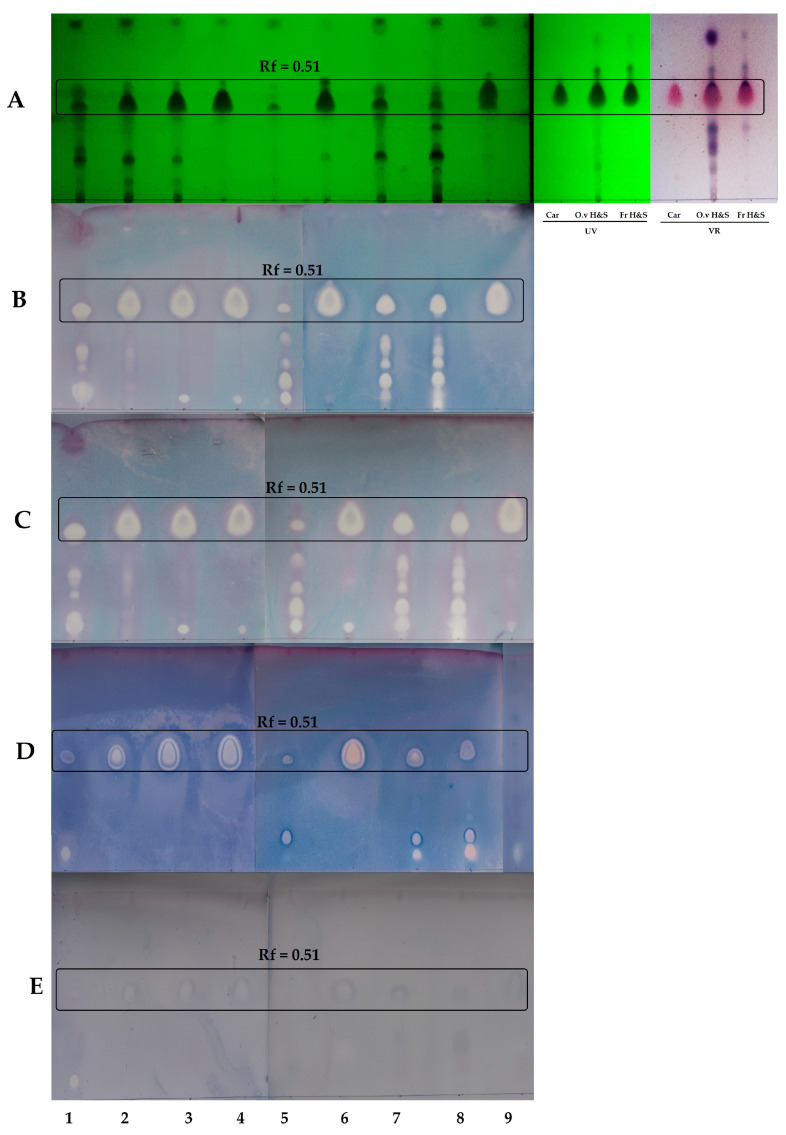
Results of thin-layer chromatography—Direct bioautography; 1 to 9—EOs’ code numbers (see Table 1), (**A**)—TLC plate photographed under UV light λ = 254 nm, (**B**)—*H. influenzae*, (**C**)—*H. parainfluenzae*, (**D**)—MRSA, (**E**)—*P. aeruginosa*, Rf—Retardation factor, VR—Vanillin reagent, Car—carvacrol reference, O.v H&S—*O. vulgare* ‘Hot & Spicy’ EO, Fr H&S—Fraction of *O. vulgare* EO with carvacrol. Adsorbent, silica gel 60 F254. Solvent, toluene–ethyl acetate, 95 + 5 (*v*/*v*).

The antibacterial effects of nine oregano essential oils were investigated using the TLC-bioautographic method. EOs after TLC separation were tested against four bacterial strains: *Staphylococcus aureus* (MRSA), *Haemophilus influenzae*, *H. parainfluenzae,* and *Pseudomonas aeruginosa*. The results are shown in Figure 1. 

Data presented in Figure 1A confirmed the results obtained by GC-MS that carvacrol is the monoterpene present in all *Origanum* EOs. Since carvacrol and thymol are a pair of isomers, they could be identified on the TLC plate as a single zone at *R*_f_ = 0.51. For EO **9**, in which both compounds were detected, thymol was the major component, and the shape of the zone at *R*_f_ = 0.51 was different in comparison to the other EOs, indicating the presence of thymol.

Figure 1B-E shows the antibacterial activity of the examined EOs against four bacterial strains. This activity was related to the most abundant component-carvacrol, but the effect of the minor components should also be taken into consideration. The plates treated with both *Haemophilus* strains (Figure 1B,C) showed the highest number of single inhibition zones. The zone at *R*_f_ = 0.51 corresponding to carvacrol also showed an antibacterial effect against methicillin-resistant *S. aureus* (Figure 1D). A few more active zones were also apparent on the TLC plates of the EOs classified in the terpineol/sabinene hydrate chemotype (EO 5, 7, 8), indicating that some more polar components were active against MRSA. The weakest activity was observed for *P. aeruginosa* (Figure 1E).

Further studies (minimum inhibitory concentration (MIC) assay and anti-biofilm assay) were conducted using essential oils that were obtained in sufficient quantities. These were EOs from the carvacrol chemotype: *O. vulgare* ‘Hirtum’ (**2**), *O. vulgare* ‘Margarita’ (**3**), *O. vulgare* ‘Hot & Spicy’ (**4**), and *O. syriacum* (I) (**6**); the terpineol/sabinene hydrate chemotype: *O. majorana* (**5**); and the thymol chemotype: *O. syriacum* (II) (**9**).

### 2.3. Minimum Inhibitory Concentration Assay

As mentioned, the minimum inhibitory concentration assay was performed for EOs 2, 3, 4, 5, 6 and 9. The MIC values against *Haemophilus influenzae*, *H. parainfluenzae*, *Pseudomonas aeruginosa*, and *Staphylococcus aureus* (MRSA) were determined using the broth microdilution assay in the concentration range of 0.0781–5 mg/mL. The data are presented in Table 2.

The results showed that the most active essential oils obtained from the aerial parts of *Origanum majorana* (**6**) were classified within the terpineol/sabinene hydrate chemotype. Compared to the other EOs, this oil showed coherently higher activity against all bacterial strains. 

All examined EOs showed the same activity against *H. influenzae* and *H. parainfluenzae* strains with MICs of 0.15 mg/mL. Therefore, these two strains were the most susceptible. *S. aureus* (MRSA) was the most “resistant” to all EOs and the MIC values were 0.6 mg/mL for the EOs belonging to the carvacrol and thymol chemotypes, except for the *O. majorana* EO (MIC = 0.3 mg/mL). The MIC values of EOs against *P. aeruginosa* were 0.3 mg/mL and only *O. majorana* EO exhibited any inhibitory effect against *P. aeruginosa* at 0.15 mg/mL. 

### 2.4. Anti-Biofilm Assay

**Figure 2:**
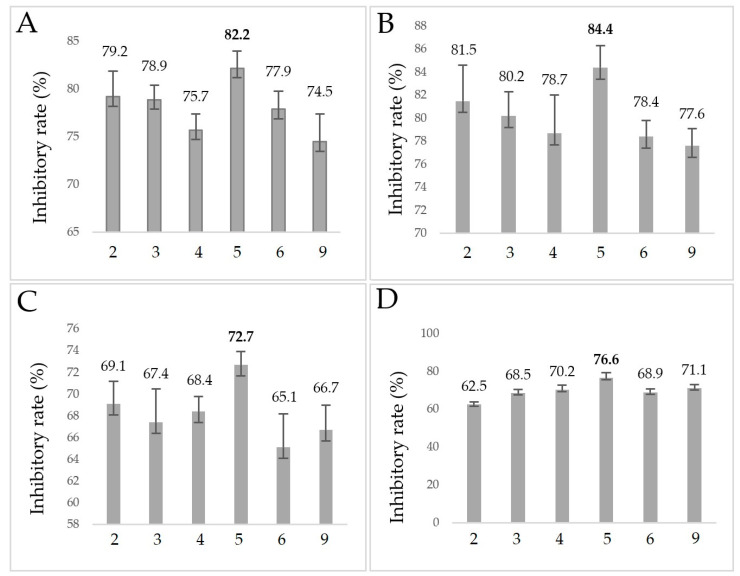
Inhibitory rates of essential oils in the biofilm assay. *O. vulgare* “Hirtum” (**2**), *O. vulgare* “Margarita” (**3**), *O. vulgare* “Hot & Spicy” (**4**), *O. majorana* (**5**), *O. syriacum* (I) (**6**), *O. syriacum* (II) (**9**); (**A**): *H. influenzae*, (**B**): *H. parainfluenzae*, (**C**): *P. aeruginosa*, (**D**): MRSA; Inhibitory rate = (1–S/C) × 100%, where C and S were defined as the average absorbance of control and sample groups, respectively.

The EOs described in Section 2.3 were also subjected to an anti-biofilm assay and the results are presented in Figure 2. 

The inhibitory rate of all EOs for *H. influenzae* and *H. parainfluenzae* was higher than that for *P. aeruginosa* or methicillin-resistant *S. aureus*, and the difference was approximately 10%. *O. majorana* EO (**5**) showed the strongest inhibition among the tested oregano Eos. In the case of the *Haemophilus* strains, *O. majorana* EO produced the highest inhibitory rates (82.2% and 84.4%). The second most effective oil against all of the tested bacteria was *O. vulgare* “Hirtum”.

### 2.5. Scanning Electron Microscopy

**Figure 3:**
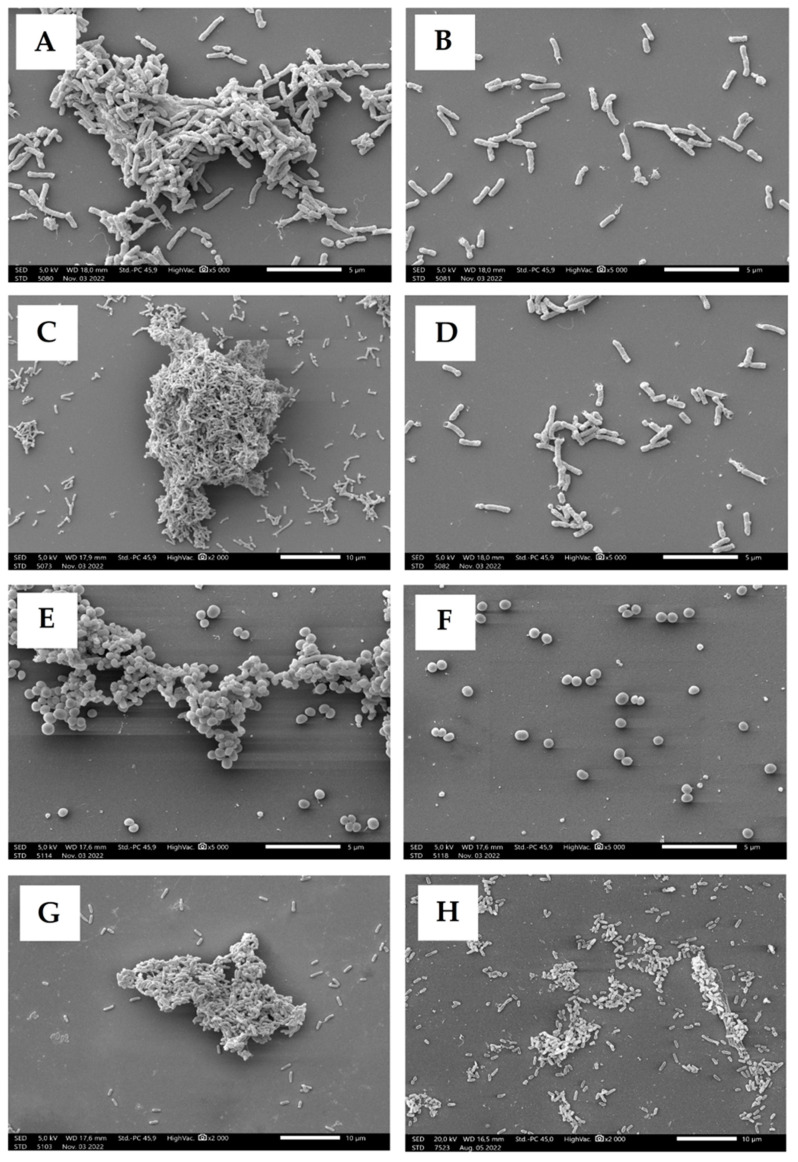
Scanning electron microscopy photographs of examined strains, control samples: (**A**)—*H. influenzae*, (**C**)—*H. parainfluenzae*, (**E**)—MRSA, (**G**)—*P. aeruginosa*. Samples treated with EO of *O. majorana* (**5**): (**B**)—*H. influenzae*, (**D**)—*H. parainfluenzae*, (**F**)—MRSA, (**H**)—*P. aeruginosa*

Scanning electron microscopy (SEM) was used to investigate the structural changes of biofilms after the EO treatment. Only the most effective EO obtained from *O. majorana* (**5**) was included in this experiment and the results are presented in Figure 3. 

Every untreated strain developed a biofilm, and these are shown in Figure 3A (*H. influenzae*), Figure 3C (*H. parainfluenzae*)*,* Figure 3E (MRSA), and Figure 3G (*P. aeruginosa*). Following EO treatment, each bacterial strain was unable to form the meshed biofilm structure and the bacteria remained discrete (Figure 3B,D,F,H). The built-up three-dimensional structure of the biofilm can be observed in the control samples (Figure 3A,C,E,G), but in case of the treated samples, no mature biofilm formed. In the case of the *Haemophilus* strains, it was observed that the adhesion of the bacterial cells took place, but the process of biofilm formation could not start. For the MRSA strain, separate planktonic cells were detected. *P. aeruginosa* was the most resistant bacterium during the anti-biofilm assay, and the SEM photographs also supports this result. In case of this strain, it was observed that the cell network of the biofilm began to form after 4 h. The embeddedness of bacterial cells in the alginate mucus can be seen in the picture. However, the mature biofilm did not form due to the treatment with EO.

## 3. Discussion

There are many different interpretations as to how pathogenic bacteria become sensitive to EOs and, therefore, their antibacterial activity is still not fully understood. Some papers suggest that this mechanism depends on the varied composition of EOs, while others focus on the activity of the major compounds alone. Among the properties that are supposed to enhance the susceptibility of pathogens to EOs, an increased fluidity of the cell membrane, a perturbed metabolism, and their ability to limit cell division are mentioned. In general, the antimicrobial activity of EOs is probably a resultant of all mentioned mechanisms [32,33,34,35]. The research of Zouhir et al. and Uzair et al. showed the need to study mixtures of different EOs or EOs in combination with antibiotics or other active components as the mixtures often exhibit synergistic bactericidal effects [36,37,38]. Due to the rich chemical composition of most EOs, there is very little chance for pathogenic bacteria to acquire resistance against them, although different strains exhibit various susceptibilities [36,39,40]. 

Three out of four strains in this study (*Haemophilus* spp. and *P. aeruginosa*) are Gram-negative bacteria, known for their increased resistance to antibiotics [41,42]. There is evidence of significant activity of EOs against these bacteria in the literature, which support the results of this work, where the two most sensitive strains were *H. influenzae* and *H. parainfluenzae* [43,44,45,46,47]. Huang et al. proved that *Artemisia asiatica* EO was active against *H. influenzae* with an MIC of 1.9 mg/mL, and SEM photographs showed changes in the bacterial cells. The major components were found to be oxygenated: piperitone, (*Z*)-davanone, and 1,8-cineole. In this study, the Gram-positive MRSA showed the strongest lack of susceptibility in the MIC assay, while other research indicated that this strain is sensitive to many EOs and the carvacrol chemotype was mentioned as significantly active [12,27,30,48]. On the other hand, in the TLC-DB assay of this work, MRSA had the largest inhibition zones at *R*_f_ = 0.51, identified as carvacrol. 

The antimicrobial activity of *Origanum* spp. EOs is widely described in the literature. Researchers examined the sensitivity of pathogenic strains such as *S. epidermidis*, *E. coli*, *S. aureus*, *S. mutans*, *Pseudomonas aeruginosa*, *Proteus vulgaris*, *Citrobacter koseri*, and *Klebsiella pneumoniae* [49,50,51,52]. Kozics et al. showed that the EO of *O. vulgare* of the carvacrol chemotype was one of the most active of the examined EOs and surprisingly significantly more active compared to cefuroxime against *P. aeruginosa*. Shamseddine et al. examined the EO of *O. syriacum* of the carvacrol chemotype and found significant inhibitory effects on *S. aureus* and *Streptococcus mutans* and that this oil could have utility in the field of dentistry. Taleb et al. studied the bactericidal effect of the thymol EO chemotype of *O. vulgare* and concluded that it was the most active against acne strains: *S. epidermidis* and *P. acne.* These results were in agreement with the high activity of carvacrol chemotype EOs examined in this work. 

The ability to create a biofilm is not only a feature of pathogenic strains but in case of their virulence, it makes them significantly less sensitive to antibiotics. Quorum sensing is a phenomenon of chemical communication between bacterial cells consisting of the synthesis and secretion of signal molecules which participate in the regulation of various physiological processes [17]. Substances that are able to disturb this signaling can enhance the susceptibility of biofilm-producing strains to antibiotics and such anti-quorum sensing activity is called quorum quenching [19,20,53]. Kalia et al. gave an evidence of such activity of cinnamon EO against *P. aeruginosa,* where both the ability to create a biofilm and extracellular secretion were inhibited [54]. Sharifi et al. examined the anti-biofilm and anti-quorum sensing activity of *Thymus daenensis* and *Satureja hortensis* EOs against *Staphylococcus aureus* isolates and concluded that disruption of the biofilm formation was observed at sub-MIC concentrations and that one of the most abundant major compounds of the EOs was carvacrol [55]. In our study, after *cis*-sabinene hydrate and terpinen-4-ol, carvacrol was the third most-abundant compound identified in the *O. majorana* EO examined in the anti-biofilm assay, and its activity could not be readily associated with the activity of the whole EO. Nevertheless, the composition of this particular EO suggests that anti-biofilm activity might depend on a mixture of major compounds. The SEM photographs presented here show visible effects on the biofilm inhibition by *O. majorana* EO against all strains. This confirmed the validity of research that combined the major compounds of the EOs into more active mixtures. Ramos et al. investigated the chemical composition and antibacterial activity of the *Origanum majorana* EO and showed that *trans*-sabinene hydrate and terpinen-4-ol were the main compounds, which is in agreement with the results presented here [56]. Several studies support the fact that EOs are proven to be more effective compared to the activity of the major components because synergism can be observed so the minor components present in the EO strengthen the effect of the main compound [57,58,59]. 

The novelty of the results presented in this work focus on comparing the activity of the *cis*-sabinene hydrate chemotype with the well-described properties of the carvacrol chemotype. The EO of *O. majorana* showed the highest activity in the MIC and anti-biofilm assays, which indicated that this chemotype represents an interesting direction for further investigation.

Interestingly, all of the nine examined *O. vulgare* cultivars could not be included into one chemotype. *O. vulgare* ‘Variegata’ and *O. vulgare* ‘Aureum’ showed the highest share of *trans*-sabinene hydrate despite having high amounts of carvacrol and, therefore, were included in a different chemotype than carvacrol. Baj et al. [9] examined the chemical composition of *Origanum* spp. EOs, where *O. vulgare* ‘Hot & Spicy’ and *O. vulgare* ‘Hirtum’ were identified as the carvacrol chemotype and *O. vulgare* ‘Aureum’ as the sabinene chemotype, which is in agreement with the results in this work. However, the composition of the two examined EOs of *O. vulgare* and the one EO of *O. majorana* in the Baj and coworkers [9] assay were different compared to the results presented here. These data suggest that, despite cultivating the same cultivars, multiple factors can affect the final chemical composition of EOs.

## 4. Materials and Methods

### 4.1. Essential Oils

Essential oils were obtained from 9 different *Origanum* species and cultivars: *Origanum vulgare* (**1**), *O. vulgare* ‘Hirtum’ (**2**), *O. vulgare* ‘Margarita’ (**3**), *O. vulgare* ‘Hot & Spicy’ (**4**), *O. majorana* (**5**), *O. syriacum* (I) (**6**), *O. vulgare* ‘Variegata’ (**7**), *O. vulgare* ‘Aureum’ (**8**), and *O. syriacum* (II) (**9**). Oreganos were cultivated in the garden of the Research and Science Innovation Center in Wola Zadybska near Lublin (Poland 51°44′49″ N 21°50′38″ E). The aerial parts were collected in June 2018, and authentication was performed by A. Kiełtyka-Dadasiewcicz. Voucher specimens were deposited in the Research and Science Innovation Center. The essential oils of air-dried plant materials were obtained by hydro-distillation for 2 h in a Deryng-type apparatus. The oils were stored in tightly sealed 1.5 mL amber vials at 4 °C prior to analysis. 

### 4.2. Gas Chromatography-Mass Spectrometry

A Shimadzu GC-2010 Plus instrument coupled to a Shimadzu QP2010 Ultra mass spectrometer (Shim-pol, Warsaw, Poland) was used for GC-MS analyses. The chromatograph was equipped with a fused-silica capillary column ZB-5 MS (30 m, 0.25 mm i.d.) with a film thickness of 0.25 mm (Phenomenex, Torrance, CA, USA). The oven temperature program was started at 50 °C, held for 3 min, then increased at the rate of 8–250 °C/min, and held for a further 2 min. The MS was operated in EI mode; the scan range was 40–500 amu, the ionization energy was 70 eV, and the scan rate was 0.20 s per scan. The injector (250 °C), interface (250 °C), and ion source (220 °C) temperatures were set. Split injection was performed with a split ratio of 1:20. Helium was the carrier gas at a 1.0 mL/min flow rate. Each of the 9 EOs samples were prepared by diluting 2 µL of EO in 1 mL of hexane. An internal standard was added to each sample. Three parallel measurements were done. The relative percentages of each component present in the EOs were calculated. The retention indices were determined in relation to a homologous series of *n*-alkanes (C_8_–C_24_) under the same operating conditions. The compounds were identified with computer-assisted spectral libraries (MassFinder 2.1 Hamburg, Germany; NIST 2011, Gaithersburg, MD, USA).

### 4.3. Cultivation of Bacterial Strains

The antibacterial effects of all investigated EOs were examined on *Haemophilus* spp., (*H. influenzae* DSM 4690; *H. parainfluenzae* DSM 8978), *Pseudomonas aeruginosa* ATCC 27853 and methicillin-resistant *Staphylococcus aureus* (MRSA, ATCC 700698). For the microbiological assays, *Haemophilus* spp. bacterial cultures were grown as follows: 100 mL of brain heart infusion (BHI) broth (Sigma Aldrich Ltd., Darmstadt, Germany) with 1 mL of supplement B (Diagon Kft., Budapest, Hungary) and 15 µg/mL of NAD solution. *P. aeruginosa* an MRSA were grown with 100 mL of BHI in a shaker incubator (C25 Incubator Shaker, New Brunswick Scientific, Edison, NJ, USA) at 37 °C and at a speed of 60 rpm for 12 h [60].

### 4.4. Thin-Layer Chromatography-Direct Bioautography (TLC-DB) Assay

For the TLC-DB assay, each EO was diluted with ethanol (1:5 *v*/*v*), and then 1 µL of each sample was applied onto a silica gel plate (Merck, silica gel 60 with fluorescent indicator F254) as a single spot. The plates were eluted in a standing chromatographic chamber with an eluent composed of toluene and ethyl acetate (95:5 *v*/*v*). Each plate was photographed under UV light λ = 254 nm and then DB was performed. The DB plate was first dipped in a chamber filled with examined bacterial suspension (4 × 10^7^ CFU/mL) for a couple of seconds. Then, it was gently dried and left for 10 min of incubation at 37 °C in a high humidity chamber. After that, the plate was dipped for a couple of seconds in 3-(4,5-dimethylthiazol-2-yl)-2,5-diphenyltetrazolium bromide (MTT) aqueous solution (0.071 g/100 mL) and then dried and left in the same conditions as described above, but for an incubation period of 24 h. Places where the growth of the bacterial strain was inhibited are called inhibition zones, and they are easily spotted due to their lack of color change in contrast to the purple color of the areas with uninhibited growth.

Each inhibition zone from each DB plate was related to a corresponding retardation factor (*R*_f_) on the plate photographed under UV light. The DB plates (bioautograms) were photographed after the incubation period. In order to identify the inhibition zones at *R*_f_ = 0.51, carvacrol reference, *O. vulgare* ‘Hot & Spicy’ EO, and carvacrol fraction of *O. vulgare* ‘Hot & Spicy’ EO were developed in the same conditions as described for the DB plates. Three parallel TLC-DB measurements were made.

### 4.5. Minimum Inhibitory Concentrations and Anti-Biofilm Assay

In order to dissolve the EOs in BHI, 1% Tween 40 (Sigma Aldrich Kft., Budapest, Hungary) was used as an emulsifier for preparing the stock solution of samples. Upon further examination, the emulsified Tween 40 did not show any inhibitory effect as the control [24]. The MIC values were determined with the broth microdilution test and 96-well microtiter plates were used. From each bacterial solution of concentration 10^5^ cfu/mL, 100 μL was added to the wells. The stock solutions of the EOs were at a concentration of 5 mg/mL. Each stock solution was prepared in BHI using 1% Tween 40 as the emulsifier and a serial two-fold dilution was made up to 0.0781 mg/mL. The incubation time was 24 h at 37 °C. The absorbance was measured at 600 nm with a plate reader (BMG Labtech, SPECTROstar Nano, Budapest, Hungary). The negative control was the Tween 40 solution, and the positive control was the untreated bacterial suspension. An average of the six replicates was calculated and then the mean of the negative control was subtracted from the value obtained. An absorbance lower than 10% of the positive control samples, i.e., growth inhibition of 90% or more, was considered as the MIC value. 

The bacterial biofilms were prepared in 96-well microtiter plate as follows: 200 µL of the bacterial culture (10^8^ cfu/mL) was added into each well and then incubated for 4 h at 37 °C. The non-adherent cells were washed with physiological saline solution. The EOs were used at MIC/2 concentrations and incubated for 24 h at 37 °C. Adherent cells were fixed with methanol for 15 min and the biofilms were dyed with 0.1% crystal violet solution for 20 min, after which the redundant dye was removed. Acetic acid 33% (*w*/*w*) was added to each well. The absorbance was measured at 595 nm with a microtiter plate reader (BMG Labtech SPECTROstar Nano, Budapest, Hungary). All tests were carried out repeatedly 8 times [61]. The anti-biofilm formation activity was calculated and demonstrated in terms of the inhibitory rate according to the equation:

Inhibitory rate = (1 − S/C) × 100%

where C and S were defined as the average absorbance of the control and sample groups, respectively [62].

### 4.6. Scanning Electron Microscopy

For this investigation, the EO sample of *O. majorana* was chosen as the most effective sample from the MIC assay, as show in Section 2.3. For biofilm formation, 5 mL of the BHI culture (10^8^ cfu/mL) of *P. aeruginosa*, MRSA, *H. influenzae,* and *H. parainfluenzae* were added into a sterilized bottle. The biofilms were created on sterile, degreased, glass coverslips. In order for the biofilm formation to occur, the prepared coverslips were placed in the bacterial suspension and then incubated (4 h at 37 °C). After the incubation period, physiological saline solution was used to remove non-adherent cells. During the treatment, *O. majorana* EO was used with a concentration of MIC/2 (5 mL), and then incubated for 24 h at 37 °C degrees. When the incubation time was over, the solutions were removed from the coverslips and washed with physiological saline. Next, the samples were prepared for SEM images. In order to attach and fix the bacterial biofilms to the surface, 2.5% glutaraldehyde was used (5 mL at room temperature for 2 h). The biofilms were then dehydrated using an ascending alcohol series: 5 mL of wash with ethanol of increasing concentrations, 50, 70, 80, 90, 95, and 98%, was used at room temperature for 30 min of rinsing. The last step of the dehydration process was rinsing with 5 mL of t-butyl-alcohol and absolute ethanol solution in 1:2, 1:1, and 2:1 ratios. For each ratio, the dehydration lasted for 1 h at room temperature and then was dehydrated with absolute t-butyl alcohol for 2 h at room temperature. The samples were stored at 4 °C for 1 h and freeze-dried overnight. The samples were coated with a gold membrane and observed with a JEOL JSM IT500-HR scanning electron microscope (Jeol Ltd., Tokyo, Japan) [63].

## 5. Conclusions

The chemotypes of the carvacrol, caryophyllene oxide, terpineol/sabinene hydrate, and thymol chemotypes were recognized among the *Origanum* EOs and all major and minor compounds of these chemotypes were oxygenated, which may be of importance for their antimicrobial activity. These results showed that oregano EOs with the carvacrol chemotype were active against all of the four examined strains, although the most active was *Origanum majorana*, classified in the terpineol/sabinene hydrate chemotype, with *trans*-sabinene hydrate and 4-terpineol as the major monoterpenes. In the anti-biofilm assay, the least susceptible strains were found to be methicillin-resistant *S. aureus* and *P. aeruginosa*. The *Haemophilus* strains used in this study were the most sensitive to the EO treatment. The cultivation of EO-rich plants can affect the chemical composition of final products which are valuable and relatively safe therapeutics for the treatment of respiratory tract infections. This work proves that essential oils obtained from *Origanum* cultivated in Poland with the carvacrol or *trans*-sabinene hydrate as the main components possess an anti-biofilm effect against the respiratory tract pathogens used in this study. 

## Figures and Tables

**Table 1:** The chemical composition—relative amounts [%] of examined oregano essential oils; RI_ex_—Retention index on ZB-5MS column, RI_lit_—Retention index from the literature (MassFinder, NIST).

RI_ex_	RI_lit_	Compound	1 *	2	3	4	5	6	7	8	9
925	932	α-Thujene	-	-	-	0.1	-	-	0.2	-	0.6
933	936	α-Pinene	-	-	0.3	0.6	-	-	0.2	-	0.5
973	973	Sabinene	-	-	-	-	-	-	0.7	0.2	-
978	978	β-Pinene	-	-	-	-	-	-	0.1	-	0.1
982	962	1-Octen-3-ol	0.7	0.7	0.4	0.2	-	0.2	1.8	1.8	0.3
984	969	3-Octanone	-	0.2	0.1	-	-	-	0.2	-	-
988	987	Myrcene	-	-	0.9	1.3	-	0.4	0.3	-	1.7
998	981	3-Octanol	0.2	-	-	-	-	0.2	0.2	-	0.4
1007	1002	α-Phellandrene	-	-	-	0.1	-	-	-	-	0.2
1018	1013	α-Terpinene	-	-	0.5	0.9	0.1	0.4	0.4	-	1.9
1026	1015	*p*-Cymene	1.8	1.1	5.8	5.6	0.1	3.8	8.2	4.1	7.0
1030	1024	Limonene	-	-	0.2	0.2	-	-	0.3	0.1	0.3
1033	1025	1,8-Cineole	0.1	0.2	0.5	-	-	-	0.4	0.2	0.2
1036	1029	(*Z*)-β-Ocimene	-	-	0.2	-	-	-	0.3	-	-
1046	1041	(*E*)-β-Ocimene	-	-	0.1	-	-	-	-	-	-
1060	1051	γ-Terpinene	-	-	2.1	2.7	0.2	1.3	0.2	-	8.9
1074	1065	*cis*-Sabinene hydrate	4.5	1.0	0.6	0.5	6.4	0.6	3.9	2.6	0.9
1087	1082	α-Terpinolene	-	-	-	0.1	-	-	-	-	0.1
1101	1086	Linalool	0.3	0.3	0.2	-	3.4	-	0.2	1.8	0.8
1105	1098	*trans*-Sabinene hydrate	1.5	0.9	0.5	0.3	37.1	0.6	26.4	28.0	1.2
1129	1108	*cis*-*p*-Menth-2-en-1-ol	0.2	-	-	-	1.7	-	0.8	1.6	-
1148	1116	*trans*-*p*-Menth-2-en-1-ol	0.1	-	-	-	0.7	-	0.4	0.1	-
1152	1123	Camphor	-	0.2	-	-	-	-	0.2	0.2	-
1162	1132	Sabina ketone	0.3	-	-	-	-	-	0.2	0.9	-
1179	1150	*endo*-Borneol	-	0.4	0.2	0.1	-	0.1	0.7	0.1	0.1
1183	1156	Neomenthol	0.3	-	-	-	-	-	-	0.2	-
1186	1164	Terpinen-4-ol	2.8	0.7	0.7	0.7	19.8	0.7	9.7	4.4	0.5
1193	1169	*p*-Cymen-8-ol	0.2	-	-	-	0.2	-	0.3	0.5	-
1201	1176	α-Terpineol	0.6	0.4	0.4	0.3	5.9	0.3	1.1	1.8	0.2
1215	1193	*trans*-Piperitol	-	-	-	-	0.4	-	0.2	0.3	-
1230	1215	Thymol methyl ether	0.1	-	-	-	-	-	-	0.1	-
1240	1221	Carvacrol methyl ether	0.2	1.8	0.8	0.4	-	-	3.8	0.3	-
1248	1239	Linalyl acetate	-	-	-	-	4.5	-	-	-	0.2
1249	1242	Carvone	0.7	0.1	-	-	-	-	0.3	0.3	-
1287	1270	Bornyl acetate	-	0.1	-	-	-	-	0.2	0.2	-
1297	1289	Terpinen-4-yl acetate	-	-	-	-	0.8	-	-	-	-
1301	1290	Thymol	0.8	0.3	0.4	0.4	-	0.6	-	5.7	46.9
1313	1300	Carvacrol	22.1	57.6	71.5	81.3	8.0	86.4	26.5	15.3	25.3
1356	1342	Neryl acetate	-	-	-	-	0.3	-	-	-	-
1367	1354	Carvacryl acetate	-	0.2	0.3	0.3	-	0.3	-	-	-
1375	1362	Geranyl acetate	-	-	-	-	0.6	-	-	-	-
1382	1379	α-Copaene	0.1	-	0.1	-	-	-	-	0.3	-
1391	1386	β-Bourbonene	1.8	0.2	0.2	-	-	-	1.1	0.9	-
1394	1389	β-Elemene	0.2	-	-	-	-	-	0.2	0.1	-
1429	1420	(*E*)-β-Caryophyllene	0.6	-	1.2	1.5	1.2	2.0	-	0.6	0.9
1438	1430	β-Copaene	0.2	0.1	-	-	-	-	0.1	0.1	-
1448	1443	Aromandendrene	-	0.2	-	-	-	-	-	-	-
1466	1455	α-Humulene	0.2	-	0.2	-	-	-	-	-	-
1470	1462	*allo*-Aromadendrene	0.7	-	-	-	-	-	-	0.8	-
1499	1494	Valencene	-	0.1	0.1	-	-	-	-	-	-
1505	1496	α-Muurolene	0.2	0.3	0.3	-	-	-	-	0.4	0.2
1511	1503	β-Bisabolene	0.4	1.9	0.6	0.2	-	-	0.5	0.2	-
1522	1507	γ-Cadinene	0.5	0.5	0.2	0.1	-	-	-	0.2	-
1525	1520	δ-Cadinene	-	0.7	0.5	0.3	-	-	-	0.5	-
1591	1572	Spathulenol	11.7	1.5	0.9	0.1	1.3		5.6	1.6	-
1596	1580	Caryophyllene oxide	15.2	6.8	1.9	0.4	1.3	0.6	0.6	4.9	-
1606	1592	Viridiflorol	0.4	-	-	-	-	-	0.1	0.2	-
1625	1602	Humulene epoxide II	3.1	1.0	0.2	0.3	-	-	-	0.7	-
1669	1643	α-Cadinol	2.3	0.3	0.2	-	-	-	0.4	2.1	-
1964	1951	Hexadecanoic acid	1.3	-	-	-	-	-	-	0.2	-
2095	2096	Heneicosane	0.8	8.2	-	-	-	-	-	-	-

* Essential oils obtained from: **1**—Origanum vulgare, **2**—Origanum vulgare ‘Hirtum’, **3**—Origanum vulgare ‘Margarita’, **4**—Origanum vulgare ‘Hot & Spicy’, **5**—Origanum majorana, **6**—Origanum syriacum (I), **7**—Origanum vulgare ‘Variegata’, **8**—Origanum vulgare ‘Aureum’, **9**—Origanum syriacum (II).

**Table 2:** Minimum inhibitory concentrations (MICs) of examined oregano essential oils.

Sample Name	Minimum Inhibitory Concentrations (mg/mL)			
	*H. influenzae*	*H. parainfluenzae*	*P. aeruginosa*	MRSA
*O. vulgare* “Hirtum” (**2**)	0.15	0.15	0.3	0.6
*O. vulgare* “Margarita” (**3**)	0.15	0.15	0.3	0.6
*O. vulgare* “Hot & Spicy” (**4**)	0.15	0.15	0.3	0.6
*O. majorana* (**5**)	0.15	0.15	0.15	0.3
*O. syriacum* (I) (**6**)	0.15	0.15	0.3	0.6
*O. syriacum* (II) (**9**)	0.15	0.15	0.3	0.6

## Data Availability

Not applicable.

## Sample Availability

Samples of the compounds are available from the authors.

## References

[1] *Origanum* Tourn. ex L. http://www.worldfloraonline.org/taxon/wfo-4000027145.

[2] Sharifi-Rad M., Berkay Yılmaz Y., Antika G., Salehi B., Tumer T.B., Kulandaisamy Venil C., Das G., Patra J.K., Karazhan N., Akram M. (2021). Phytochemical constituents, biological activities, and health-promoting effects of the genus Origanum. Phyther. Res..

[3] Kaskatepe B., Erdem S.A., Ozturk S., Oz Z.S., Subasi E., Koyuncu M., Vlainić J., Kosalec I. (2022). Antifungal and Anti-Virulent Activity of *Origanum majorana* L. Essential Oil on Candida albicans and In Vivo Toxicity in the Galleria mellonella Larval Model. Molecules.

[4] Gutiérrez-Grijalva E.P., Picos-Salas M.A., Leyva-López N., Criollo-Mendoza M.S., Vazquez-Olivo G., Heredia J.B. (2018). Flavonoids and phenolic acids from Oregano: Occurrence, biological activity and health benefits. Plants.

[5] Yu H., Zhang P., Liu H., Sun X., Liang J., Sun L., Chen Y. (2021). Hypoglycemic activity of *Origanum vulgare* L. And its main chemical constituents identified with HPLC-ESI-QTOF-MS. Food Funct..

[6] Balusamy S.R., Perumalsamy H., Huq M.A., Balasubramanian B. (2018). Anti-proliferative activity of *Origanum vulgare* inhibited lipogenesis and induced mitochondrial mediated apoptosis in human stomach cancer cell lines. Biomed. Pharmacother..

[7] Abdel-Massih R.M., Fares R., Bazzi S., El-Chami N., Baydoun E. (2010). The apoptotic and anti-proliferative activity of *Origanum majorana* extracts on human leukemic cell line. Leuk. Res..

[8] Gong H.Y., Liu W.H., LV G.Y., Zhou X. (2014). Analysis of essential oils of *Origanum vulgare* from six production areas of China and Pakistan. Rev. Bras. Farmacogn..

[9] Baj T., Sieniawska E., Ludwiczuk A., Widelski J., Kiełtyka-Dadasiewicz A., Skalicka-Woźniak K., Głowniak K. (2017). Thin-layer chromatography-fingerprint, antioxidant activity, and gas chromatography-mass spectrometry profiling of several *Origanum* L. species. J. Planar Chromatogr.–Mod. TLC.

[10] Zhao Y., Yang Y.H., Ye M., Wang K.B., Fan L.M., Su F.W. (2021). Chemical composition and antifungal activity of essential oil from *Origanum vulgare* against Botrytis cinerea. Food Chem..

[11] Marchese A., Orhan I.E., Daglia M., Barbieri R., Di Lorenzo A., Nabavi S.F., Gortzi O., Izadi M., Nabavi S.M. (2016). Antibacterial and antifungal activities of thymol: A brief review of the literature. Food Chem..

[12] Suntres Z.E., Coccimiglio J., Alipour M. (2015). The Bioactivity and Toxicological Actions of Carvacrol. Crit. Rev. Food Sci. Nutr..

[13] Bassolé I.H.N., Juliani H.R. (2012). Essential oils in combination and their antimicrobial properties. Molecules.

[14] Sawicki R., Golus J., Przekora A., Ludwiczuk A., Sieniawska E., Ginalska G. (2018). Antimycobacterial activity of cinnamaldehyde in a mycobacterium tuberculosis(H37Ra) model. Molecules.

[15] Piasecki B., Biernasiuk A., Skiba A., Skalicka-Woźniak K., Ludwiczuk A. (2021). Composition, anti-MRSA activity and toxicity of essential oils from Cymbopogon species. Molecules.

[16] Chouhan S., Sharma K., Guleria S. (2017). Antimicrobial Activity of Some Essential Oils—Present Status and Future Perspectives. Medicines.

[17] Wang Y., Bian Z., Wang Y. (2022). Biofilm formation and inhibition mediated by bacterial quorum sensing. Appl. Microbiol. Biotechnol..

[18] Ciofu O., Moser C., Jensen P.Ø., Høiby N. (2022). Tolerance and resistance of microbial biofilms. Nat. Rev. Microbiol..

[19] Singh B.N., Prateeksha N., Upreti D.K., Singh B.R., Defoirdt T., Gupta V.K., De Souza A.O., Singh H.B., Barreira J.C.M., Ferreira I.C.F.R. (2017). Bactericidal, quorum quenching and anti-biofilm nanofactories: A new niche for nanotechnologists. Crit. Rev. Biotechnol..

[20] Sepahi E., Tarighi S., Ahmadi F.S., Bagheri A. (2015). Inhibition of quorum sensing in Pseudomonas aeruginosa by two herbal essential oils from Apiaceae family. J. Microbiol..

[21] Yuan J., Yuan W., Guo Y., Wu Q., Wang F., Xuan H. (2022). Anti-Biofilm Activities of Chinese Poplar Propolis Essential Oil against Streptococcus mutans. Nutrients.

[22] Guimarães Silva Vasconcelos P., Medeiros de Almeida Maia C., Mendes de Vasconcelos V., Paolla Raimundo e Silva J., Fechine Tavares J., Vieira Pereira J., Wanderley Cavalcanti Y., Maria Melo de Brito Costa E. (2022). In vitro inhibition of a multispecies oral cavity biofilm by Syzygium aromaticum essential oil. Gerodontology.

[23] Kwiatkowski P., Sienkiewicz M., Pruss A., Łopusiewicz Ł., Arszyńska N., Wojciechowska-Koszko I., Kilanowicz A., Kot B., Dołęgowska B. (2022). Antibacterial and Anti-Biofilm Activities of Essential Oil Compounds against New DelhiMetallo-β-Lactamase-1-Producing Uropathogenic *Klebsiella pneumoniae* Strains. Antibiotics.

[24] Kerekes E.B., Vidács A., Takó M., Petkovits T., Vágvölgyi C., Horváth G., Balázs V.L., Krisch J. (2019). Anti-biofilm effect of selected essential oils and main components on mono- and polymicrobic bacterial cultures. Microorganisms.

[25] Paudel P.N., Satyal P., Satyal R., Setzer W.N., Gyawali R. (2022). Chemical Composition, Enantiomeric Distribution, Antimicrobial and Antioxidant Activities of *Origanum majorana* L. Essential Oil from Nepal. Molecules.

[26] Yasar S., Nizamlloǧlu N.M., Gücüş M.O., Bildik Dal A.E., Akgül K. (2022). *Origanum majorana* L. Essential Oil-Coated Paper Acts as an Antimicrobial and Antioxidant Agent against Meat Spoilage. ACS Omega.

[27] Silva E.C.A.d., Leuthier L.L., Almeida Júnior A., Nunes J.M.F.F., Correia Sampaio F., Farias I.A.P. (2022). Physicochemical characteristics and antimicrobial activity of *Origanum vulgare* L. essential oil and carvacrol on cariogenic bacteria: An in vitro and in silico study. Nat. Prod. Res..

[28] Sarac N., Ugur A. (2008). Antimicrobial activities of the essential oils of *Origanum onites* L., *Origanum vulgare* L. subspecies hirtum (link) Ietswaart, Satureja thymbra L., and Thymus cilicicus Boiss. & Bal. growing wild in Turkey. J. Med. Food.

[29] Alma M.H., Mavi A., Yildirim A., Digrak M., Hirata T. (2003). Screening chemical composition and in vitro antioxidant and antimicrobial activities of the essential oils from *Origanum syriacum* L. growing in Turkey. Biol. Pharm. Bull..

[30] Hao Y., Li J., Shi L. (2021). A Carvacrol-Rich Essential Oil Extracted From Oregano (*Origanum vulgare* “Hot & Spicy”) Exerts Potent Antibacterial Effects Against Staphylococcus aureus. Front. Microbiol..

[31] Baranska M., Schulz H., Krüger H., Quilitzsch R. (2005). Chemotaxonomy of aromatic plants of the genus Origanum via vibrational spectroscopy. Anal. Bioanal. Chem..

[32] Wang X., Shen Y., Thakur K., Han J., Zhang J.G., Hu F., Wei Z.J. (2020). Antibacterial Activity and Mechanism of Ginger Essential Oil against Escherichia coli and Staphylococcus aureus. Molecules.

[33] Angane M., Swift S., Huang K., Butts C.A., Quek S.Y. (2022). Essential Oils and Their Major Components: An Updated Review on Antimicrobial Activities, Mechanism of Action and Their Potential Application in the Food Industry. Foods.

[34] Li Y.X., Erhunmwunsee F., Liu M., Yang K., Zheng W., Tian J. (2022). Antimicrobial mechanisms of spice essential oils and application in food industry. Food Chem..

[35] Vasconcelos N.G., Croda J., Simionatto S. (2018). Antibacterial mechanisms of cinnamon and its constituents: A review. Microb. Pathog..

[36] Zouhir A., Jridi T., Nefzi A., Ben Hamida J., Sebei K. (2016). Inhibition of methicillin-resistant Staphylococcus aureus (MRSA) by antimicrobial peptides (AMPs) and plant essential oils. Pharm. Biol..

[37] Uzair B., Niaz N., Bano A., Khan B.A., Zafar N., Iqbal M., Tahira R., Fasim F. (2017). Essential oils showing in vitro anti MRSA and synergistic activity with penicillin group of antibiotics. Pak. J. Pharm. Sci..

[38] Sharifi-Rad M., Varoni E.M., Iriti M., Martorell M., Setzer W.N., del Mar Contreras M., Salehi B., Soltani-Nejad A., Rajabi S., Tajbakhsh M. (2018). Carvacrol and human health: A comprehensive review. Phyther. Res..

[39] Trifan A., Luca S.V., Greige-Gerges H., Miron A., Gille E., Aprotosoaie A.C. (2020). Recent advances in tackling microbial multidrug resistance with essential oils: Combinatorial and nano-based strategies. Crit. Rev. Microbiol..

[40] Wińska K., Mączka W., Łyczko J., Grabarczyk M., Czubaszek A., Szumny A. (2019). Essential oils as antimicrobial agents—Myth or real alternative?. Molecules.

[41] Eichenberger E.M., Thaden J.T. (2019). Epidemiology and mechanisms of resistance of extensively drug resistant gram-negative bacteria. Antibiotics.

[42] Otsuka Y. (2020). Potent antibiotics active against multidrug-resistant gram-negative bacteria. Chem. Pharm. Bull..

[43] Balázs V.L., Horváth B., Kerekes E., Ács K., Kocsis B., Varga A., Böszörményi A., Nagy D.U., Krisch J., Széchenyi A. (2019). Anti-haemophilus activity of selected essential oils detected by TLC-direct bioautography and biofilm inhibition. Molecules.

[44] Huang J., Qian C., Xu H., Huang Y. (2018). Antibacterial activity of Artemisia asiatica essential oil against some common respiratory infection causing bacterial strains and its mechanism of action in Haemophilus influenzae. Microb. Pathog..

[45] Sela F., Karapandzova M., Stefkov G., Cvetkovikj I., Kulevanova S. (2015). Chemical composition and antimicrobial activity of essential oils of Juniperus excelsa Bieb. (Cupressaceae) grown in R. Macedonia. Pharmacognosy Res..

[46] Luciardi M.C., Blázquez M.A., Alberto M.R., Cartagena E., Arena M.E. (2020). Grapefruit essential oils inhibit quorum sensing of Pseudomonas aeruginosa. Food Sci. Technol. Int..

[47] Van L.T., Hagiu I., Popovici A., Marinescu F., Gheorghe I., Curutiu C., Ditu L.M., Holban A.M., Sesan T.E., Lazar V. (2022). Antimicrobial Efficiency of Some Essential Oils in Antibiotic-Resistant Pseudomonas aeruginosa Isolates. Plants.

[48] Horváth G., Jámbor N., Végh A., Böszörményi A., Lemberkovics É., Héthelyi É., Kovácsc K., Kocsisc B. (2010). Antimicrobial activity of essential oils: The possibilities of TLC-bioautography. Flavour Fragr. J..

[49] Taleb M.H., Abdeltawab N.F., Shamma R.N., Abdelgayed S.S., Mohamed S.S., Farag M.A., Ramadan M.A. (2018). *Origanum vulgare* L. Essential oil as a potential anti-acne topical nanoemulsion—In vitro and in vivo study. Molecules.

[50] Lagha R., Abdallah F.B., AL-Sarhan B.O., Al-Sodany Y. (2019). Antibacterial and Biofilm Inhibitory Activity of Medicinal Plant Essential Oils Against Escherichia coli Isolated from UTI Patients. Molecules.

[51] Shamseddine L., Chidiac J.J. (2021). Composition’s effect of *Origanum syriacum* essential oils in the antimicrobial activities for the treatment of denture stomatitis. Odontology.

[52] Kozics K., Bučková M., Puškárová A., Kalászová V., Cabicarová T., Pangallo D. (2019). The effect of ten essential oils on several cutaneous drug-resistant microorganisms and their cyto/genotoxic and antioxidant properties. Molecules.

[53] Falsafi T., Moradi P., Mahboubi M., Rahimi E., Momtaz H., Hamedi B. (2015). Chemical composition and anti-Helicobacter pylori effect of Satureja bachtiarica Bunge essential oil. Phytomedicine.

[54] Kalia M., Yadav V.K., Singh P.K., Sharma D., Pandey H., Narvi S.S., Agarwal V. (2015). Effect of cinnamon oil on quorum sensing-controlled virulence factors and biofilm formation in Pseudomonas aeruginosa. PLoS ONE.

[55] Sharifi A., Mohammadzadeh A., Zahraei Salehi T., Mahmoodi P. (2018). Antibacterial, antibiofilm and antiquorum sensing effects of Thymus daenensis and Satureja hortensis essential oils against Staphylococcus aureus isolates. J. Appl. Microbiol..

[56] Ramos S., Rojas L.B., Lucena M.E., Meccia G., Usubillaga A. (2011). Chemical composition and antibacterial activity of *origanum majorana* L. Essential oil from the venezuelan andes. J. Essent. Oil Res..

[57] Gencic M., Aksic J., Stosic Z., Randjelovic P., Stojanovic N., Radic Z., Radulovic N. (2021). Linking the antimicrobial and anti-inflammatory effects of immortelle essential oil with its chemical composition–The interplay between the major and minor constituents. Food Chem. Toxicol..

[58] Hsouna A., Halima N., Abdelkafi S., Hamdi N. (2013). Essential oil from Artemisia phaeolepis: Chemical composition and antibacterial activities. J. Oleo Sci..

[59] Miladinovic D., Dimitrijevic M., Krstev T., Markovic M., Ciric V. (2021). The signifcance of minor components on the antibacterial activity of essential oil via chemometrics. LWT.

[60] Humphries R.M., Hindler J.A., James H.J., Karen C.C., Guido F., Michael A.P., Marie L.L., Sandra S.R., David W.W. (2011). Susceptibility Test Methods: Fastidious Bacteria. Manual of Clinical Microbiology.

[61] Peeters E., Nelis H.J., Coenye T. (2008). Comparison of multiple methods for quantification of microbial biofilms grown in microtiter plates. J. Microbiol. Methods.

[62] Sun Y., Chen S., Zhang C., Liu Y., Ma L., Zhang X. (2018). Effects of sub-minimum inhibitory concentrations of lemon essential oil on the acid tolerance and biofilm formation of Streptococcus mutans. Arch. Oral Biol..

[63] Kerekes E.B., Deák É., Takó M., Tserennadmid R., Petkovits T., Vágvölgyi C., Krisch J. (2013). Anti-biofilm forming and anti-quorum sensing activity of selected essential oils and their main components on food-related micro-organisms. J. Appl. Microbiol..

